# Implementation of Text-Messaging and Social Media Strategies in a Multilevel Childhood Obesity Prevention Intervention: Process Evaluation Results

**DOI:** 10.1177/0046958018779189

**Published:** 2018-06-04

**Authors:** Ivory H. Loh, Teresa Schwendler, Angela C.B. Trude, Elizabeth T. Anderson Steeves, Lawrence J. Cheskin, Sarah Lange, Joel Gittelsohn

**Affiliations:** 1 Johns Hopkins Bloomberg School of Public Health, Baltimore, MD, USA; 2 Peace Corps, The Gambia; 3 The University of Tennessee, Knoxville, USA; 4 Johns Hopkins Weight Management Center, Baltimore, MD, USA

**Keywords:** process evaluation, social media, text messaging, obesity, evaluation studies, health promotion, social marketing, Baltimore, African Americans, caregivers

## Abstract

Social media and text messaging show promise as public health interventions, but little evaluation of implementation exists. The B’more Healthy Communities for Kids (BHCK) was a multilevel, multicomponent (wholesalers, food stores, recreation centers) childhood obesity prevention trial that included social media and text-messaging components. The BHCK was implemented in 28 low-income areas of Baltimore City, Maryland, in 2 waves. The texting intervention targeted 241 low-income African American caregivers (of 283), who received 3 texts/week reinforcing key messages, providing nutrition information, and weekly goals. Regular posting on social media platforms (Facebook, Instagram, Twitter) targeted community members and local stakeholders. High implementation standards were set a priori (57 for social media, 11 for texting), with low implementation defined as <50%, medium as 50% to 99%, high as ≥100% of the high standard for each measure. Reach, dose delivered, and fidelity were assessed via web-based analytic tools. Between waves, social media implementation improved from low-moderate to high reach, dose delivered, and fidelity. Text messaging increased from moderate to high in reach and dose delivered, fidelity decreased from high to moderate. Data were used to monitor and revise the BHCK intervention throughout implementation. Our model for evaluating text messaging–based and social media–based interventions may be applicable to other settings.

**What do we already know about this topic?**
  Past interventions have successfully tested social media and text messaging’s positive effect on healthy eating, weight loss, and physical activity, but few have set evaluation metrics for these strategies.
**How does your research contribute to the field?**
  This paper sets process evaluation standards for implementation of a social media and text-messaging program in a multilevel-multicomponent obesity prevention intervention and suggests best practices for managing these platforms.
**What are your research’s implications toward theory, practice, or policy?**
  This research suggests the benefit of conducting detailed process evaluation when aiming to improve intervention implementation and provides practices that researchers can use in future interventions to promote behavior change through social media and text messaging.

## Introduction

Obesity is a public health crisis with significant costs for individuals, communities, and governments.^1,2^ In Baltimore, the obesity epidemic affects adults and children, particularly black and lower income populations with less access to healthful foods.^3,4^

As there are various factors that contribute to childhood obesity, research has urged for multilevel interventions that simultaneously address these factors.^5,6^ Multilevel-multicomponent (ML-MC) interventions intervene at multiple settings concurrently (eg, corner stores, worksites, and recreation centers) and use multiple components (eg, education, social marketing, policy) to target sustainable systems change.^7^ However, these programs can be costly to implement and may have weak reach to target audiences due to diffusion of limited resources.^7,8^ Multiple intervention strategies need to connect and reinforce each other, such that intervention components are synchronized across levels.^7^

Social media and text messaging have the potential to support multilevel interventions due to their high reach, relatively low cost, and ability to tie together diverse interventions.^9,10^ Internet and social media use have substantially risen over the past decade, with 65% of American adults using at least 1 social media site as of 2015.^11^ Social media refers to Internet-based platforms that allow people to communicate and interact.^10^ Text messaging is another common form of communication across all age groups; more than 70% of cell phone owners in the age groups 18 to 29 years, 30 to 49 years, and 50 to 64 years cited that they used their phones to send or receive text messages in 2013.^12^ African Americans and Hispanics are more likely to text than their white counterparts.^12^ Although several interventions have successfully tested social media and text messaging’s positive effect on healthy eating,^9^ weight loss,^13^ and physical activity,^14,15^ few have set evaluation metrics for these strategies, which detracts from our understanding and use of these technologies as intervention tools.^14,16^ In addition, we have not identified any study to date that has set standards for implementation of social media and text messaging in a ML-MC obesity prevention intervention. This paper addresses these gaps by describing the process evaluation of the text messaging and social media components of the B’more Healthy Communities for Kids (BHCK) intervention.^17^

## Methods

### Intervention Overview

The BHCK was a childhood obesity prevention, group-randomized trial implemented in 2 waves.^17^ In each wave, 14 zones were selected upon meeting study’s eligibility criteria: low-income, predominantly African American (>50%) neighborhood with a recreation center at least 0.5 miles from a supermarket. Neighborhoods were randomized to be an intervention or comparison zone (n = 28 total zones). Different zones were used for waves 1 and 2. Within each zone, an evaluation sample of African American caregivers, defined as any adult (>18 years old) responsible for taking care of a 10- to 14-year-old child, was actively recruited at community sites (eg, recreation centers, corner stores, carryout restaurants, parks). The first wave of BHCK intervention was implemented from June 2014 to February 2015; the second wave from November 2015 to July 2016.^17^ Our evaluation sample included 150 and 133 caregivers in wave 1 and wave 2, respectively. The BHCK intervened on different components of the Baltimore food environment (corner stores, carryout restaurants, wholesaler, and recreation centers) and at different socioecological levels (policy, small food stores, afterschool program, and informational environment—social media and text messaging).

Prior to intervention, three focus groups with youth, ages 10 to 14 years, and their adult caregivers were conducted in predominantly African American communities in Baltimore (n = 20). Most participants used at least one of the following social media sites: Facebook, Twitter, Instagram, or Tumblr, with Facebook being most popular. Participants were open to communicating and receiving BHCK updates through social media and texts, and provided feedback on the types of messages they preferred to receive.

Based on the formative research, we chose Facebook, Instagram, Twitter, and text messaging to reach caregivers and connect and reinforce various BHCK intervention components. Content delivered on these platforms paralleled the 3 phases delivered in other intervention levels: smart drinks, smart snacks, and smart cooking. Intervention was guided by social cognitive theory, with goal-setting messages and bidirectional interactions to enhance self-efficacy and reinforce positive health behaviors to encourage behavioral change.^18^ Sharing recipes and individual stories of success on our social media accounts and text-messaging campaign promoted observational learning. Replying encouraging messages to BHCK participants who texted us with questions or responses to our challenge-of-the-week text messages also promoted behavioral reinforcement. The BHCK interventionists also sought to improve self-efficacy with supportive posts that helped people to identify where healthier choices were available in their food environment, especially when promoted healthy food and drink items were stocked and on sale in the corner stores and carryout restaurants that were cooperating with BHCK.

The Johns Hopkins Bloomberg School of Public Health Institutional Review Board (IRB No. 00004203) approved this research; written informed consent was obtained from all adult participants.

### Social Media and Text-Messaging Recruitment

Wave 1 recruitment for social media and text messaging started 1 month before overall BHCK intervention in June 2014 to build initial follower base. All eligible caregivers in the intervention evaluation arm (n = 150) were invited to join via email, phone, and letter using contact information gathered at their baseline interview.^17^ Participants could choose between receiving 3 and 5 texts per week. They joined the text-messaging program with a specific keyword and were invited to follow BHCK social media pages.

Wave 2 recruitment of caregivers in the evaluation intervention sample (n = 133) took place during the baseline interview (April-November 2015). Those who were not invited or initially declined invitation were called afterward about enrolling in the program. Based on wave 1 participants’ preference for 3 texts per week, all wave 2 participants were texted 3 texts per week at off-peak hours.

To involve the community, social media handles were printed on all wave 2 posters and handouts distributed at stores and recreation centers, and on cooperating carryout menus. Participants in the intervention evaluation arm were actively invited to follow the pages through email, mail, texts, and calls. Unlike text messaging, social media pages were open to the public to reach a larger audience and potential stakeholders.

### Text-Messaging Intervention

The BHCK text-messaging intervention targeted adult caregivers. Messages were drafted by BHCK interventionists, who were undergraduate and graduate students trained in nutrition and public health, revised by the registered dietitians on the research team, approved by the primary investigator. The content was designed to encourage bidirectional communication and allowed for individual follow-up (Supplemental Table S1). Contact names were used to personalize text messages. First text of the week was goal-oriented, with the promoted task related to the unit concurrently taught at the recreation center and corner stores. To encourage response from participants, a *yes* or *no* question was typically sent at the end of the week, asking whether they achieved the weekly challenge. Other message content included information to support goal attainment, nutrition information, recipes for promoted food items, and their cost and availability at corner stores (Supplemental Table S1). Enrolled participants could opt-out at any point.

*TextIt* (https://textit.in) was used in wave 1, because it allowed scheduling of message flows, with automated responses to preset questions. Due to issues with message delivery and scheduling inflexibility, BHCK switched to *EZ Texting* (https://app.eztexting.com) during wave 2. The BHCK also used *MobileVip* (http://mobilevip.me), a custom-made texting platform that allowed scheduling of automated responses to preset questions and improved successful text message delivery.

### Social Media Intervention

Social media, namely Facebook, Instagram, and Twitter, broadened BHCK by targeting the whole Baltimore community. Content promoted other program components, including BHCK posters and handouts, photos from recreation centers and corner store interventions, the youth leaders (local college students trained to deliver the BHCK afterschool program), and cooperating wholesalers and corner storeowners. The BHCK social media also shared Baltimore-specific news and community events to engage the community.

#### Facebook

It allowed for longer and more frequent posts than text messaging and the other social media platforms (ie, Instagram and Twitter) and use of links, photos and videos, creating a more comprehensive communication platform to reach study sample and other members within and beyond Baltimore. The wave 1 Facebook page was maintained for wave 2 to retain initial follower base. Using a set weekly posting schedule (Supplemental Table S2), interventionists posted at least once a day in the evening, which was when most of BHCK’s followers were online based on *Facebook Analytics*, to optimize post engagement.

#### Instagram

During wave 1, users were less engaged with the “BHCK1” Instagram page than our Facebook Page, such that the Instagram page had much fewer followers and likes and comments per post. A new account with a catchier title of “Bmore4kids” was hence created for wave 2. Before wave 2 implementation, preintervention posting about general nutrition and networking (via liking, following, and commenting) with users within the target community helped create a substantial follower base (n = 1443) with the aim of giving credence to the account as a reliable source for nutrition advice and community news. The BHCK interventionists posted according to a weekly posting schedule (Supplemental Table S3) with 1 to 2 posts per day, as posting on average 1.5 times per day was found to be best practice.^19^ All Instagram posts were generally linked to and posted on Facebook. The BHCK had weekly Instagram challenges that corresponded to that week’s text-messaging goal, and large giveaway challenges per phase to increase participation. Large giveaway challenges were different to the weekly challenges in that they are larger scale, with the competition open for entry for longer than a week and involved gift incentives, such that all Baltimore residents who participated in the giveaway would be entered into a random drawing for a prize.

#### Twitter

In wave 1, Twitter was used to connect with caregivers and shared similar content to Facebook and Instagram. However, we found poor engagement, as local caregivers were not active Twitter users. The BHCK team learned Baltimore policy makers and organizations were more active on Twitter, so a new Twitter page, “Bmore4kids,” was created for wave 2. It focused on connecting with agencies that had an interest in changing the food environment and supplemented the BHCK policy component.

#### Boosting and use of hashtags

To increase reach, BHCK paid to “boost” 1 to 2 posts per week on Facebook near the end of wave 1 and on all social media platforms during wave 2. “Boosted” posts display content to a broader preselected audience: adults ages 18 to 65 in Baltimore City. The BHCK interventionists also boosted its overall Facebook page and tracked both paid and organic, meaning via unpaid distribution, average reach (ie, number of people who saw any activity from the page) per month. Hashtags are prevalently used on social media, especially Twitter and Instagram, to categorize and help users find content. Ten BHCK hashtags were created with 2 hashtags per phase based on the 3 phase slogans used on BHCK communication materials (eg, #RefreshBHCK #SnackSmartBHCK) (Supplemental Table S4).

### Definitions of Key Process Indicators

Prior to intervention, process evaluation standards were created to track implementation quality and make improvements during the program. Multiple reach, dose delivered, and fidelity indicators were initially developed based on previous intervention experience,^20^ and applied to each social media platform and text messaging, reviewed and refined as needed during BHCK team meetings (bimonthly). *Reach* was defined as the number of followers and impressions, ie, the number of times the page or post was displayed, or percentage of families in the evaluation sample enrolled in the text-messaging program. *Dose delivered* accounted for the number of BHCK posts on social media platforms and text messages sent. *Fidelity* was the effectiveness of the text-messaging and social media components, and measured indirectly through level of engagement that the page, posts, and texts generated (eg, likes, comments, replies), and follower retention rate.

### Process Evaluation Standards

Reach, dose delivered, and fidelity were measured by 20 preset standards in wave 1 and an expanded set of 68 standards in wave 2 (Supplemental Tables S5-S7). The BHCK set standards based on current literature,^13,21^ and measurement data available from social media analytical sites. Each standard could be met at a low, medium, or high level, with high being the goal to reflect optimum intervention delivery according to BHCK initial plans. Aiming to improve implementation for wave 2, ranges for low, medium, and high were increased (ie, high ranges in wave 1 standards were set as moderate for wave 2). The BHCK also modified its social media standards based on lessons learned and experience from wave 1. Due to the different process evaluation standards used between waves 1 and 2, this paper evaluates implementation of both waves using wave 2 standards. Impact results for the BHCK intervention are presented elsewhere.^22^

### Data Sources and Analysis

*TextIt* (wave 1), *EZ Texting*, and *MobileVip* (wave 2) were online platforms used to send messages and track process data (ie, reach, dose, and fidelity). During wave 1, *Facebook Insights, Instagram*, and *HootSuite* were used to track follower growth and posts on Facebook, Instagram, and Twitter, respectively. For wave 2, *Iconosquare* was used for Instagram and *Twitter Analytics* for Twitter. Data were measured on a weekly and monthly basis and reported as an average at the end of each phase.

Process data were derived from various program applications and recorded in Microsoft Word and Excel 2011. Process evaluation data for each platform were computed separately. All dose, reach, and fidelity standards were calculated as a percentage of the high standard met so that results would be comparable with similar interventions. We defined *low, medium*, and *high* as *<50%, 50% to 99%*, and *100% or above of the high minimum standard*, respectively. Standard percentages were averaged to calculate total reach, total dose delivered, and total fidelity for a total wave 1 average across the phases and an average for each of the 3 BHCK phases in wave 2 to track the progress of wave 2 implementation against wave 1.

## Results

### Reach

#### Text messaging

**Figure 1.**
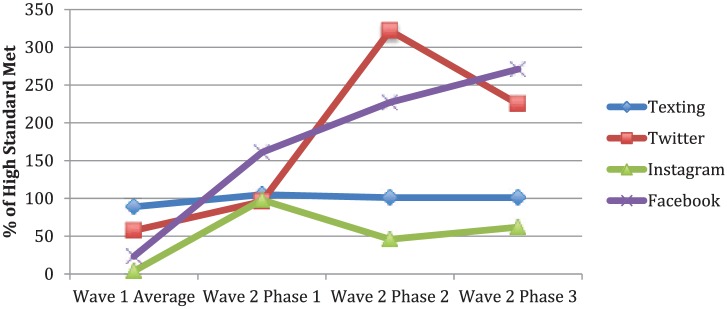
Social media and text-messaging intervention reach. *Note.* The data points were calculated by averaging the percentage of minimum high standard met for all reach standards. Implementation was considered low for <50% of the high standard. Medium is 50% to 99% of the high standard, high is 100% or above of the high standard. For texting, 2 out of 2 of wave 2 reach standards were analyzed for both wave 1 and wave 2. Based on wave 2 Facebook standards, 1 out of 6 reach standards were analyzed for wave 1; six out of 6 reach standards for wave 2. Based on wave 2 Instagram standards, 1 out of 2 reach standards were analyzed for wave 1, and 2/2 for wave 2. For Twitter, 1/6 wave 2 reach standards were analyzed for wave 1 and 6/6 for wave 2.

Based on wave 2 standards, total reach of the wave 1 text-messaging program was moderate (Figure 1). Of the entire intervention evaluation sample, 97% of all caregivers (n = 150) were successfully invited to join the BHCK texting program through email, phone call, or letter, with 5 participants unable to be reached due to invalid phone number, email, or home address. Seventy-two percent (n = 108) of the wave 1 caregivers enrolled. In wave 2, of 133 caregivers in the intervention group, 82% enrolled in the texting, reflecting high reach.

#### Social media

In wave 1, low to moderate reach was achieved for new page likes (Facebook) or follower growth (Instagram, Twitter) per month (Table 1). Over the course of wave 2, Facebook, Instagram, and Twitter gained an average of 285.5, 403.7, and 105 new page likes or followers per month, respectively, reflecting high reach. By intervention’s end, Instagram built the largest follower base (n = 4317), followed by Facebook and Twitter (Supplemental Table S8). Wave 2 overall reach for Facebook and Twitter was consistently high—an improvement over the total moderate reach for both platforms during wave 1. Instagram improved from low to moderate total reach, meeting 62% of high minimum standard on average across the 3 phases as compared with 3.8% achieved in wave 1.

**Table 1.** Summary of Dose Delivered, Reach, and Fidelity Measurements for Social Media and Text Messaging in Wave 1 and Wave 2 by Wave 2 Standards.

Social media process measure	Wave 1 average	Wave 2 average	Platform for data collection
Facebook			
Reach^a^			
1. No. of new Facebook page likes/month	Low	High	Facebook Analytics
2. Average no. of paid total reach/month	NC	High	
3. Average no. of organic total reach/month	NC	High	
Dose delivered			
1. No. of posts made per week on Facebook	Medium	High	Facebook Analytics
2. No. of Facebook boosts per week	High	High	
Fidelity			
1. Average no. of shares/month	NC	High	Facebook Analytics
2. Average no. of comments by participants/post per month	NC	Medium	
3. Average no. of reactions/post per month	NC	High	
Instagram			
Reach			
1. No. of people reached per Instagram campaign	NC	Low	Iconosquare
2. No. of new followers/month	Low	Medium	
Dose delivered			
1. No. of media posted/week	Medium	High	Iconosquare
2. Average no. of hashtags per posts	NC	High	
3. No. of large Instagram challenges/phase	NC	Medium	
Fidelity			
1. Total no. of likes on posts by month	NC	High	Iconosquare
2. Total no. of comments on posts by month	NC	High	
Twitter			
Reach			
1. Total impressions/week	NC	High	Twitter Analytics
2. No. of net follower growth/week	Medium	High	
Dose delivered			
1. No. of tweets posted/day	Medium	Medium	Twitter Analytics
2. No. of retweets made per week about our followers by BHCK team	NC	High	
Fidelity			
1. No. of likes received/week	NC	Medium	Twitter Analytics
2. No. of retweets/week	NC	High	
3. No. of link clicks/week	NC	High	
Text-messaging process measure			
Reach			
1. Percentage of families sign up for text messaging	Medium	High	Microsoft Excel Records
2. Percentage of BHCK enrolled families receive an invitation to join the text-messaging program	Medium	High	
Dose			
1. No. of text messages are sent to all participants each week	High	High	EZ Texting Records
2. No. of goal-setting text messages per week	Medium	Medium	
Fidelity			
1. Percentage of families enrolled who stayed enrolled in the program for at least 2 months	High	High	Interventionist Records MobileVip Records
2. Percentage of families enrolled who stay enrolled in the program for at least 6 months	Low	Medium	
3. Percentage of text messages responses received from participants when questions are prompted	Medium	Low	

*Note.* BHCK = B’more Healthy Communities for Kids. NC = Data Not Collected.

a Total reach is the number of unique people who saw any activity from BHCK page, including posts, others’ post on BHCK page, page like ads, mentions, and check-ins. Organic reach is the number of unique people who saw BHCK post through unpaid distribution, whereas paid reach is the total number of people who saw BHCK post as a result of advertisements.^25^

### Dose Delivered

#### Text messaging

**Figure 2.**
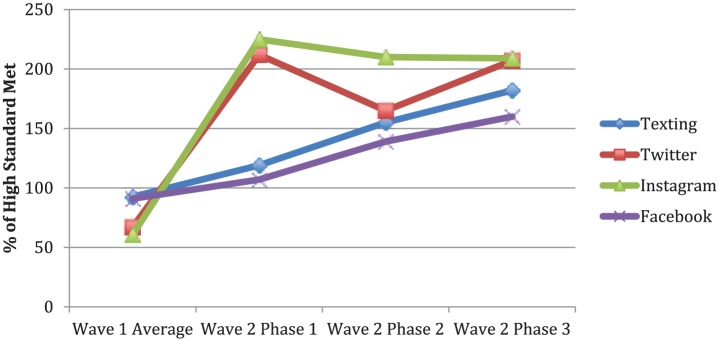
Social media and text-messaging intervention dose delivered. *Note.* The data points were calculated by averaging the percentage of minimum high standard met for all dose delivered standards. Low dose delivery was defined as meeting <50% of high standard. Moderate dose delivered was 50% to 99%, and high dose delivered is ≥100%. For texting, 6 out of 7 wave 2 dose delivered standards were analyzed for wave 1 and 7 out of 7 for wave 2. For Twitter, 2/5 wave 2 dose delivered standards were analyzed for wave 1 and 5/5 for wave 2; for Instagram, 1/9 collected for wave 1 and 9/9 for wave 2; for Facebook, 2/7 collected for wave 1 and 7/7 for wave 2.

On average, wave 1 participants in the 2-texts per week group received 2.3 texts per week, and participants in the 3-to-5 texts per week group received 3.1 texts per week. Moderate total dose delivered was achieved in wave 1 (Figure 2), as goal-setting text messages were only sent on 10 of 17 weeks. Wave 2 participants received 4.5 texts per week and 1 goal-setting text weekly, achieving high total dose delivered. Use of *MobileVip* also improved text delivery during wave 2, averaging 89.5% and 91.3% successfully delivered texts per week in phases 2 and 3.

#### Social media

During wave 1, 9.3, 10.5, and 7.6 posts per week were made on average to Facebook, Instagram, and Twitter, respectively, with 18.6, 7.2, and 7.4 posts per week during wave 2. New social media dose standards were developed and met during wave 2 to assess delivery of different post types (ie, video, article discussion, youth leader feature) to ensure variety and improve engagement. Total dose delivered improved from moderate to high from wave 1 to 2 across all platforms (Figure 2).

### Fidelity

#### Text messaging

**Figure 3.**
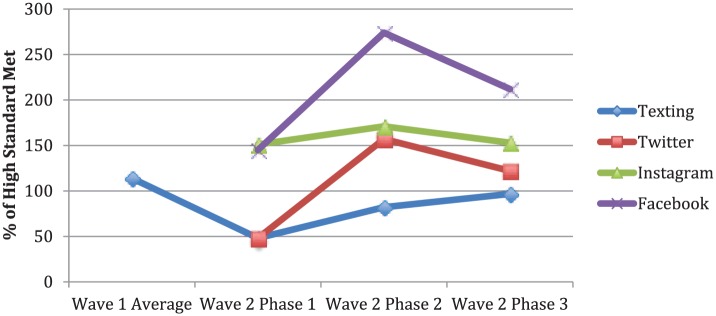
Social media and text-messaging intervention fidelity. *Note.* The data points were calculated by averaging the percentage of minimum high standard met for all fidelity standards. Low fidelity was defined as meeting <50% of high standard, 50% to 99% (moderate), and ≥100% high. For texting, 4/4 wave 2 fidelity standards were analyzed for both waves 1 and 2. For all social media, no wave 2 fidelity standard was collected during wave 1. Six out of 6 wave 2 Twitter fidelity standards were collected for wave 2; nine out of nine for Instagram; five out of five for Facebook.

Total fidelity for wave 1 text messaging was high (Figure 3). Participation retention rate was high, with 87% of initially enrolled caregivers (n = 94) remaining for at least 6 months (Supplemental Table S8). On average, 26.9% of participants responded when questions were prompted. Wave 2 texting achieved moderate fidelity with an averaged 9.5% weekly response rate (Table 1). Overall, 59.5% of families (n = 105) stayed enrolled in the program for at least 6 months, while 96.3% of families remained for at least 2 months. This discrepancy is largely because many participants were interviewed and invited into the text-messaging program after overall start of intervention, so they were not yet enrolled for 6 months by intervention end. Opt-out rate declined across the 3 phases in wave 2, from 12.1% to 3.1% and 0%.

#### Social media

Fidelity was newly defined after wave 1 with new wave 2 standards set to indirectly measure fidelity through engagement. Thus, no data were collected for these standards in wave 1, and social media fidelity was not quantified. Fidelity in wave 2 was consistently high for Facebook, Instagram, and Twitter (Table 1). On average, Facebook gained 19 shares, 66.3 reactions, and 3.8 comments per post per month. Instagram received 60.8 likes and 1.5 comments per post. Twitter received 34.2 page mentions per month and 67.9 page likes per week.

## Discussion

To our knowledge, this is the first community-based obesity prevention trial to include multiple social media platforms and text messaging as part of a multi-level, multi-component intervention, and to report on implementation quality. Overall, social media implementation improved from low-to-moderate to high reach, dose delivered, and fidelity between the 2 waves. Text-messaging implementation improved to high reach and dose delivered.

A novel component of this study was its measurement of fidelity. Fidelity is typically defined as quality of delivery—or a more refined version of dose delivered.^23^ We chose to assess fidelity indirectly, as engagement in the target population. We quantified engagement by number of likes, comments, and shares associated with each post. This approach has been used by others.^13^ Engagement has also been measured in other ways, including post views^21^ and link clicks,^10^ which we instead defined as reach. We deemed engagement as an action on the viewer’s part and used it to assess fidelity, as social media and texting were created for social networking. Although engagement assessed for BHCK social media pages included individuals beyond our target audience (as pages were open to the general public), an overall higher engagement level would still reflect improvement in the intervention’s fidelity.

The BHCK interventionists found that posting timely and personalized content (ie, cultural-specific and community-specific posts, individualized text responses) and encouraging goal-setting and discussion were successful ways to promote each platform. These techniques were also shown to be successful in previous mobile-based^14,24^ and social media interventions.^21^

Beyond boosting to increase reach, social networking was most effective at increasing reach and gaining followers. Throughout wave 2, BHCK interventionists dedicated time to like and retweet from relevant Twitter accounts, and join Twitter chats. The BHCK also made “shout-out” posts for other accounts on Instagram and Facebook, to feature weekly challenge winners and connect with other users. Dose delivered standards were set in wave 2 to track these activities.

Linking social media pages also helped increase reach by spreading messages to a greater audience. The BHCK Instagram posts were always linked to Facebook and often to Twitter. The BHCK interventionists observed increases in likes and comments on linked posts. However, effectiveness of different media types varies within platforms. For instance, hyperlinks cannot be shared on Instagram; Facebook followers were less likely to watch a video if a link was posted instead of the video itself.

A limitation found in this study and previous studies^16^ was that the quantifiable engagement with social media posts and text messages was not always directly correlated with behavioral change. Including specific measures for behavior changes within intervention may help, such as asking users to post a photo of their home-cooked meal, which BHCK did as part of its large Instagram challenges.

Another challenge encountered was in assessing percentage of the evaluation sample participating in BHCK social media sites due to individuals’ privacy settings and use of different usernames. To overcome this, BHCK tried to gauge engagement by using example posts in the dose received (“exposure”) evaluation during post-intervention assessment with study participants.

The BHCK texting response rate was low compared with another study that received an average 68.5% response rate to self-monitoring text messages.^13^ Difference in response rates was likely due to the sample population age, as their participants were college students, who are probably more familiar with texting than BHCK’s older caregiver sample (mean age = 41 years). However, like the study by Napolitano et al, BHCK participants also reported positive feedback about receiving health-related text messages.

Finally, changes in standards between waves 1 and 2 prevented comparison between some process evaluation standards. However, new process evaluation measures were added based on best practices and lessons learned in wave 1, and ultimately improved evaluation and implementation during wave 2.

## Conclusions

The BHCK showed that social media and text messaging were innovative tools to include in and increase reach of a multilevel community intervention. Valuable improvements in intervention implementation made in wave 2, based on lessons learned from wave 1, suggest the benefit of implementing the program in 2 waves and conducting detailed process evaluation. The overall high level of reach and fidelity achieved by end of intervention demonstrated the need for consistent high dose delivery and community interest in using social media and text messages to receive and engage with health-related topics. This study shows the importance of understanding the target audience’s usage of social media (ie, which platform(s), frequency, peak hours) to maximize engagement and successfully utilize it as an intervention tool. Recommended practices for managing social media sites include boosting, networking with other accounts, and posting varied and population-relevant content consistently. In the text-messaging program, use of personalized texts, goal-oriented messages, and yes-or-no prompt questions were among the recommended strategies. This work furthers the field by identifying features that increase engagement and retention of target audience and detailing an effective implementation plan and process evaluation standards for social media and text messaging. Future researchers can apply these practices in their own interventions to promote behavior change and improve reach.

## Supplementary Material

Supplementary Material, Supplemental_Table_S1 – Implementation of Text-Messaging and Social Media Strategies in a Multilevel Childhood Obesity Prevention Intervention: Process Evaluation Results Click here for additional data file. Supplementary Material, Supplemental_Table_S1 for Implementation of Text-Messaging and Social Media Strategies in a Multilevel Childhood Obesity Prevention Intervention: Process Evaluation Results by Ivory H. Loh, Teresa Schwendler, Angela C.B. Trude, Elizabeth T. Anderson Steeves, Lawrence J. Cheskin, Sarah Lange, and Joel Gittelsohn in INQUIRY: The Journal of Health Care Organization, Provision, and Financing

## Supplementary Material

Supplementary Material, Supplemental_Table_S2 – Implementation of Text-Messaging and Social Media Strategies in a Multilevel Childhood Obesity Prevention Intervention: Process Evaluation Results Click here for additional data file. Supplementary Material, Supplemental_Table_S2 for Implementation of Text-Messaging and Social Media Strategies in a Multilevel Childhood Obesity Prevention Intervention: Process Evaluation Results by Ivory H. Loh, Teresa Schwendler, Angela C.B. Trude, Elizabeth T. Anderson Steeves, Lawrence J. Cheskin, Sarah Lange, and Joel Gittelsohn in INQUIRY: The Journal of Health Care Organization, Provision, and Financing

## Supplementary Material

Supplementary Material, Supplemental_Table_S3 – Implementation of Text-Messaging and Social Media Strategies in a Multilevel Childhood Obesity Prevention Intervention: Process Evaluation Results Click here for additional data file. Supplementary Material, Supplemental_Table_S3 for Implementation of Text-Messaging and Social Media Strategies in a Multilevel Childhood Obesity Prevention Intervention: Process Evaluation Results by Ivory H. Loh, Teresa Schwendler, Angela C.B. Trude, Elizabeth T. Anderson Steeves, Lawrence J. Cheskin, Sarah Lange, and Joel Gittelsohn in INQUIRY: The Journal of Health Care Organization, Provision, and Financing

## Supplementary Material

Supplementary Material, Supplemental_Table_S4 – Implementation of Text-Messaging and Social Media Strategies in a Multilevel Childhood Obesity Prevention Intervention: Process Evaluation Results Click here for additional data file. Supplementary Material, Supplemental_Table_S4 for Implementation of Text-Messaging and Social Media Strategies in a Multilevel Childhood Obesity Prevention Intervention: Process Evaluation Results by Ivory H. Loh, Teresa Schwendler, Angela C.B. Trude, Elizabeth T. Anderson Steeves, Lawrence J. Cheskin, Sarah Lange, and Joel Gittelsohn in INQUIRY: The Journal of Health Care Organization, Provision, and Financing

## Supplementary Material

Supplementary Material, Supplemental_Table_S5 – Implementation of Text-Messaging and Social Media Strategies in a Multilevel Childhood Obesity Prevention Intervention: Process Evaluation Results Click here for additional data file. Supplementary Material, Supplemental_Table_S5 for Implementation of Text-Messaging and Social Media Strategies in a Multilevel Childhood Obesity Prevention Intervention: Process Evaluation Results by Ivory H. Loh, Teresa Schwendler, Angela C.B. Trude, Elizabeth T. Anderson Steeves, Lawrence J. Cheskin, Sarah Lange, and Joel Gittelsohn in INQUIRY: The Journal of Health Care Organization, Provision, and Financing

## Supplementary Material

Supplementary Material, Supplemental_Table_S6 – Implementation of Text-Messaging and Social Media Strategies in a Multilevel Childhood Obesity Prevention Intervention: Process Evaluation Results Click here for additional data file. Supplementary Material, Supplemental_Table_S6 for Implementation of Text-Messaging and Social Media Strategies in a Multilevel Childhood Obesity Prevention Intervention: Process Evaluation Results by Ivory H. Loh, Teresa Schwendler, Angela C.B. Trude, Elizabeth T. Anderson Steeves, Lawrence J. Cheskin, Sarah Lange, and Joel Gittelsohn in INQUIRY: The Journal of Health Care Organization, Provision, and Financing

## Supplementary Material

Supplementary Material, Supplemental_Table_S7 – Implementation of Text-Messaging and Social Media Strategies in a Multilevel Childhood Obesity Prevention Intervention: Process Evaluation Results Click here for additional data file. Supplementary Material, Supplemental_Table_S7 for Implementation of Text-Messaging and Social Media Strategies in a Multilevel Childhood Obesity Prevention Intervention: Process Evaluation Results by Ivory H. Loh, Teresa Schwendler, Angela C.B. Trude, Elizabeth T. Anderson Steeves, Lawrence J. Cheskin, Sarah Lange, and Joel Gittelsohn in INQUIRY: The Journal of Health Care Organization, Provision, and Financing

## Supplementary Material

Supplementary Material, Supplemental_Table_S8 – Implementation of Text-Messaging and Social Media Strategies in a Multilevel Childhood Obesity Prevention Intervention: Process Evaluation Results Click here for additional data file. Supplementary Material, Supplemental_Table_S8 for Implementation of Text-Messaging and Social Media Strategies in a Multilevel Childhood Obesity Prevention Intervention: Process Evaluation Results by Ivory H. Loh, Teresa Schwendler, Angela C.B. Trude, Elizabeth T. Anderson Steeves, Lawrence J. Cheskin, Sarah Lange, and Joel Gittelsohn in INQUIRY: The Journal of Health Care Organization, Provision, and Financing
