# Supplementary material for: Implementation of Text-Messaging and Social Media Strategies in a Multilevel Childhood Obesity Prevention Intervention: Process Evaluation Results
Source: Inquiry. 2018 Jun 4;55:0046958018779189. doi: 10.1177/0046958018779189 (PMC6022210; doi:10.1177/0046958018779189)
Supplement: Supplementary Material, Supplemental_Table_S1 – Implementation of Text-Messaging and Social Media Strategies in a Multilevel Childhood Obesity Prevention Intervention: Process Evaluation Results [file Supplemental_Table_S1.pdf]

Supplemental Table S1: Representative weekly sequencing of SMS Messages and Sample Texts*

	**Sunday**	**Wednesday**	**Friday**
**Text Type**	Weekly Topic Goal	Weekly Topic Tip	Goal Follow-up Question
**Sample Text Message**	Want ur kids to eat more fruit? Leave bananas, apples, or oranges on the dining table for them to grab a sweet snack after school. Try it at least 1x this wk!	Tip from a Bmore parent w/ picky eaters: Cut up ur kids' fav fruits into small pieces & mix w/ nuts and seeds or blend w/ yogurt for a nutritious smoothie!	Fruits is an anytime and any day snack! Did u or ur kids try snacking on fruits at least 1x this week?<br><br>*If Response is Yes:*<br>That's awesome! Fruits and vegetables are the perfect #Smart snack to #refuel your body with. Keep encouraging ur kids and family to snack on them!<br><br>*If Response is No:*<br>That's alright! Fruits and vegetables are the perfect #Smart snack to #refuel your body with. Try encouraging ur kids and family to snack on them next week!

*On average, 3 texts were sent every week to participants at 7pm. Participants would receive another text if he/she responded to the yes or no SMS question and/or texted additional questions or comments to BHCK.
