# Supplementary material for: Implementation of Text-Messaging and Social Media Strategies in a Multilevel Childhood Obesity Prevention Intervention: Process Evaluation Results
Source: Inquiry. 2018 Jun 4;55:0046958018779189. doi: 10.1177/0046958018779189 (PMC6022210; doi:10.1177/0046958018779189)
Supplement: Supplementary Material, Supplemental_Table_S2 – Implementation of Text-Messaging and Social Media Strategies in a Multilevel Childhood Obesity Prevention Intervention: Process Evaluation Results [file Supplemental_Table_S2.pdf]

Supplemental Table S2: Facebook Weekly Posting Schedule for BHCK Wave 2

**Day of the Week**	**Type of Post**
Sunday	Discussion prompting question or poll
Monday	Recipe of a Healthy Dish, typically one that uses our promoted food item
Tuesday	Article link related to our phase, general nutrition or current health news in Baltimore
Wednesday	Video relevant to our phase<br><br>Eg. Cooking video of a healthy recipe, one of the BHCK videos shown to store owners or at recreation centers
Thursday	Picture relevant to the phase;<br>Bi-weekly Youth Leader feature
Friday	Fun Fact Friday with #FunFactFridayBHCK in caption
Saturday	Link/recipe/video with content similar to the ones posted on the other days
Anytime	Instagram picture linked to Facebook<br>Real time notification of interactive session or relevant local event
