# Supplementary material for: Implementation of Text-Messaging and Social Media Strategies in a Multilevel Childhood Obesity Prevention Intervention: Process Evaluation Results
Source: Inquiry. 2018 Jun 4;55:0046958018779189. doi: 10.1177/0046958018779189 (PMC6022210; doi:10.1177/0046958018779189)
Supplement: Supplementary Material, Supplemental_Table_S3 – Implementation of Text-Messaging and Social Media Strategies in a Multilevel Childhood Obesity Prevention Intervention: Process Evaluation Results [file Supplemental_Table_S3.pdf]

Supplemental Table S3: Instagram Weekly Posting Schedule for BHCK Wave 2

Day of the Week	Type of Post
Sunday	Challenge for the week with #SundayChallengeBHCK in caption<br><br>Every phase, there will be 2-3 large challenges with gift prizes that replaces one of these mini weekly challenges.
Monday	Inspiration quote or motivational picture
Tuesday	Food picture relevant to our phase with its recipe in the caption if relevant
Wednesday	Picture relevant to general nutrition
Thursday	Picture related to another component of the study and a shout out post of another account, typically an account from Baltimore
Friday	Picture or video relevant to our phase
Saturday	Feature of winner to the weekly challenge with #SpotlightSaturdayBHCK in caption
