# Supplementary material for: Implementation of Text-Messaging and Social Media Strategies in a Multilevel Childhood Obesity Prevention Intervention: Process Evaluation Results
Source: Inquiry. 2018 Jun 4;55:0046958018779189. doi: 10.1177/0046958018779189 (PMC6022210; doi:10.1177/0046958018779189)
Supplement: Supplementary Material, Supplemental_Table_S4 – Implementation of Text-Messaging and Social Media Strategies in a Multilevel Childhood Obesity Prevention Intervention: Process Evaluation Results [file Supplemental_Table_S4.pdf]

**Supplemental Table S4: BHCK Hashtags* Created Per Phase**

| **Phase and Slogan** | **BHCK Hashtag** |
|---|---|
| Smart Drinks<br><br>*Refresh with a Smart Drink* | #RefreshBHCK<br><br>#DrinkSmartBHCK |
| Smart Snacks<br><br>*Refuel with a Smart Snack* | #RefuelBHCK<br><br>#SnackSmartBHCK |
| Smart Cooking<br><br>*Reach your potential with Smart Cooking* | #CookSmartBHCK<br><br>#ReachYourPotentialBHCK |
| Anytime | #Bmore4kids<br><br>#SundayChallengeBHCK<br><br>#SpotlightSaturdayBHCK<br><br>#FunFactFridayBHCK |

*BHCK hashtags were used on all social media platforms and text messages.
