# Supplementary material for: Implementation of Text-Messaging and Social Media Strategies in a Multilevel Childhood Obesity Prevention Intervention: Process Evaluation Results
Source: Inquiry. 2018 Jun 4;55:0046958018779189. doi: 10.1177/0046958018779189 (PMC6022210; doi:10.1177/0046958018779189)
Supplement: Supplementary Material, Supplemental_Table_S5 – Implementation of Text-Messaging and Social Media Strategies in a Multilevel Childhood Obesity Prevention Intervention: Process Evaluation Results [file Supplemental_Table_S5.pdf]

Supplemental Table S5: Process Evaluation Standards for Reach on Social Media and Text Messaging during Wave 2

**Facebook**	**Low**	**Med**	**High**
Average # of paid total reach[i]/month	<400	400-600	600+
Average # of organic total reach[i]/month	<100	100-200	200+
# of new page likes/month	<100	100-200	>200
% of our people reached from Baltimore taken per month	<70%	70-85%	>85%
% of our fans from Baltimore taken per month	<70%	70-85%	>85%
**Texting**	**Low**	**Med**	**High**
% of families sign up for text-messaging	<70%	70-80%	>80%
% of BHCK enrolled families receive an invitation to join the text-messaging program	<80%	80-90%	100%
**Twitter**	**Low**	**Med**	**High**
Total Impressions[iii]/week	<5,000	5,000 – 15,000	>15,000
Weekly Impressions from Campaigns (Boosts)[iv]	<7,500	7,500-10,000	>10,000
# new twitter followers/month	<10	10 to 20	>20
# net follower growth/ week	0-3	3 to 5	≥6
# of New Followers from Campaigns (Boosts)/week	<5	5 to 10	>10
# of Profile visits/month	<540	540-750	>750
**Instagram**	**Low**	**Med**	**High**
# of people reached per Campaign (Boost)[v]	<20,000	20,00-25,000	>25,000
# of new followers/month	<300	300-450	>450

[i] Paid reach is the total number of unique people that saw a boosted post through as a result of ads

[ii] Organic reach is the number of unique people that saw a post through unpaid distribution

[iii] Twitter defines impression to be the number of times your content was seen, versus the number of people who have seen your content (reach).

[iv] Twitter Campaigns are equivalent to Facebook boosts, in that it is a paid post that can appear higher up on your target audience's NewsFeed

[v] Instagram Campaigns, equivalent to Facebook Boosts, and paid posts that are promoted on Instagram to a target audience of the user's choosing
