# Supplementary material for: Implementation of Text-Messaging and Social Media Strategies in a Multilevel Childhood Obesity Prevention Intervention: Process Evaluation Results
Source: Inquiry. 2018 Jun 4;55:0046958018779189. doi: 10.1177/0046958018779189 (PMC6022210; doi:10.1177/0046958018779189)
Supplement: Supplementary Material, Supplemental_Table_S6 – Implementation of Text-Messaging and Social Media Strategies in a Multilevel Childhood Obesity Prevention Intervention: Process Evaluation Results [file Supplemental_Table_S6.pdf]

Supplemental Table S6: Process Evaluation Standards for Dose Delivered for Social Media and Text Messaging during Wave 2

Facebook	Low	Med	High
# of discussion forum/polls per month	0-1	2 to 3	4+
# of videos per month	0-1	2 to 3	4+
# of photos per month	<15	15-29	30+
# of post about other levels (carry out, rec, stores, policy etc.) per month	<15	15-29	30+
# of posts featuring youth leaders per month	0-2	3 to 5	6+
# posts made per week on Facebook	<8	8 to 13	14+
# of Facebook boosts per week	0	1	≥2
BHCK staff will make changes to content of social media posts based on assessment of popular posts. Assessments will be completed at least once every two weeks	<2 weeks	once every 2 weeks	once every week
**Texting**	**Low**	**Med**	**High**
Average % per week of successful deliveries per text message to each participant group	<75%	75 – 90%	>90%
# of text message that link to other social media/phase	0	1-2	≥3
# text-messages are sent to all participants each week	0-1	2	≥3
# of goal setting text messages per week	0	0-0.99	≥1
BHCK staff monitors/checks text messages received from participants at least 3 times per week during the intervention	<3	3 – 5	>5
**Twitter**	**Low**	**Med**	**High**
# of twitter accounts BHCK follows/month	<25	25-49	50+
Average # of hashtags per post	N/A	0	≥1
# tweets made per week about BHCK project activities	0-1	2	≥3
# tweets made per week about our policy-related activities	0	1	≥2
# retweets made per week about our followers (related to diet, exercise, nutrition, community, farms, stores, health, etc.)	0-2	3 to 6	≥6
BHCK staff monitor/check tweets made by our followers at least 3 times per week during the intervention	<3	3 – 5	>5
# of Tweets posted/day	<5	5 to 10	>10
**Instagram**	**Low**	**Med**	**High**
# of large Instagram challenges/phase	0	1	≥2
# of weekly Instagram campaign per phase	<4	4 to 6	>6
# of shoutout/feature posts made/month	0-1	2	≥3
# of target audience that we follow/month	<2	3 to 5	>5
% of posts with our specific # per phase by month	<70%	70-90%	>90%
Average # of hashtags per posts	<5	5 to 8	≥9
# of media posted/week	<4	4 to 6	≥7
# of different types of posts made per week (e.g. types: video, feature, recipe, picture)	<2	2 to 4	≥5

# of linkages to other components (rec, carry out, corner stores) of the study made per week	0	1	≥2
