# Supplementary material for: Implementation of Text-Messaging and Social Media Strategies in a Multilevel Childhood Obesity Prevention Intervention: Process Evaluation Results
Source: Inquiry. 2018 Jun 4;55:0046958018779189. doi: 10.1177/0046958018779189 (PMC6022210; doi:10.1177/0046958018779189)
Supplement: Supplementary Material, Supplemental_Table_S7 – Implementation of Text-Messaging and Social Media Strategies in a Multilevel Childhood Obesity Prevention Intervention: Process Evaluation Results [file Supplemental_Table_S7.pdf]

Supplemental Table S7: Process Evaluation Standards for Fidelity for Social Media and Text Messaging during Wave 2

**Facebook**	**Low**	**Med**	**High**
Average # of shares/month	0-1	2 to 4	5+
Average # of reactions/post per month	0-6	7 o 14	15+
Average # of comments by participants/post per month	0-1	2 to 3	4+
Average total # of responses to discussions received per month	<4	4 to 6	7+
# of posts about BHCK made by the youth leaders per month	<3	4 to 7	>7
**Text Messaging**	**Low**	**Med**	**High**
% of families enrolled who stay enrolled in the program for at least 2 months	<50%	50 – 75%	>75%
% of families enrolled who stay enrolled in the program for at least 4 months	<50%	50 – 75%	>75%
% of families enrolled who stay enrolled in the program for at least 6 months	<50%	50 – 75%	>75%
% of text messages responses received from participants when questions are prompted	<15%	15 – 30%	≥30%
**Twitter**	**Low**	**Med**	**High**
# of Mentions received/month	<10	10 to 20	>20
# of Likes received / week	<35	35-70	>70
# of Link Clicks / week	<10	10 to 20	>20
# of Retweets / week	<10	10 to 20	>20
# of Replies / week	<5	5 to 10	>10
# of Engagements from Campaigns/Week	<120	120-175	>175
**Instagram**	**Low**	**Med**	**High**
# of our hashtags made by others/phase	<20	20-40	>40
# of posts about BHCK made by the Youth leaders per phase	<20	20-39	40+
# of entries to bigger Instagram challenges per phase	<20	20-40	>40
# of link clicks received per Instagram campaign	<50	50-75	>75
# of likes received per Instagram campaign post	<175	175-200	>200
Total # of likes on posts by month	<200	200-299	300+
Average # of likes per post	<20	20-34	35+
Total # of comments on posts by month	<10	10 to 15	15+
Average # of comments per post	<3	3 to 6	7+
