# Supplementary material for: Implementation of Text-Messaging and Social Media Strategies in a Multilevel Childhood Obesity Prevention Intervention: Process Evaluation Results
Source: Inquiry. 2018 Jun 4;55:0046958018779189. doi: 10.1177/0046958018779189 (PMC6022210; doi:10.1177/0046958018779189)
Supplement: Supplementary Material, Supplemental_Table_S8 – Implementation of Text-Messaging and Social Media Strategies in a Multilevel Childhood Obesity Prevention Intervention: Process Evaluation Results [file Supplemental_Table_S8.pdf]

Supplemental Table 8: Social Media and Text-Messaging Enrollment by the end of Wave 1 and Wave 2

Platform	Wave 1	Wave 2
Facebook*	333 Page Likes/Fans	2,946 Page Likes/Fans
Instagram	137 Followers	4,317 Followers
Twitter	70 Followers	694 Followers
Text-Messaging	108 Enrolled (72%)	109 Enrolled (82%)
*The same Facebook page was maintained from Wave 1 to Wave 2, while new accounts were made for all other social media platforms.		
